# *tert*-Butyl 4-(4-chloro­anilino)-6-methyl-2-oxocyclo­hex-3-ene­carboxyl­ate

**DOI:** 10.1107/S1600536810051743

**Published:** 2010-12-24

**Authors:** Mariano S. Alexander, Henry North, Kenneth R. Scott, Ray J. Butcher

**Affiliations:** a Department of Pharmaceutical Sciences, Howard University, 2300 4th Street NW, Washington, DC 20059, USA; b Department of Chemistry, Howard University, 525 College Street NW, Washington, DC 20059, USA

## Abstract

In the title compound, C_18_H_22_ClNO_3_, the dihedral angle between the benzene ring and the conjugated part of the enaminone ring is 55.19 (9)°. The ester substituent makes a dihedral angle of 81.0 (2)° with this latter moiety. The crystal structure features N—H⋯O and weak C—H⋯O inter­molecular inter­actions.

## Related literature

Our research on enamino­nes has led to several compounds possessing anti­convulsant properties, see: Edafiogho *et al.* (1992 ▶); Eddington *et al.* (2003 ▶); Scott *et al.* (1993 ▶, 1995 ▶).
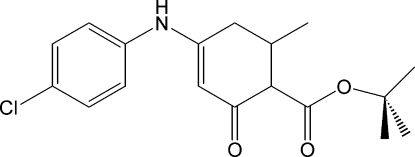


## Experimental

### 

#### Crystal data

C_18_H_22_ClNO_3_
*M* *_r_* = 335.82
Orthorhombic, 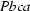

*a* = 11.0801 (3) Å
*b* = 10.9095 (3) Å
*c* = 29.2474 (7) Å
*V* = 3535.39 (16) Å^3^
*Z* = 8
Mo *K*α radiation
μ = 0.23 mm^−1^
*T* = 295 K
0.45 × 0.38 × 0.08 mm

#### Data collection

Oxford Diffraction Xcalibur Ruby Gemini diffractometer
Absorption correction: multi-scan (*CrysAlis PRO*; Oxford Diffraction, 2007 ▶) *T*_min_ = 0.93, *T*_max_ = 0.98
9798 measured reflections
3708 independent reflections
2925 reflections with *I* > 2σ(*I*)
*R*_int_ = 0.030

#### Refinement

*R*[*F* ^2^ > 2σ(*F* ^2^)] = 0.055
*wR*(*F* ^2^) = 0.172
*S* = 1.09
3708 reflections
212 parameters
H-atom parameters constrained
Δρ_max_ = 0.39 e Å^−3^
Δρ_min_ = −0.25 e Å^−3^

### 

Data collection: *CrysAlis PRO* (Oxford Diffraction, 2007 ▶); cell refinement: *CrysAlis PRO*; data reduction: *CrysAlis PRO*; program(s) used to solve structure: *SHELXS97* (Sheldrick, 2008 ▶); program(s) used to refine structure: *SHELXL97* (Sheldrick, 2008 ▶); molecular graphics: *SHELXTL* (Sheldrick, 2008 ▶); software used to prepare material for publication: *SHELXL97*.

## Supplementary Material

Crystal structure: contains datablocks I, global. DOI: 10.1107/S1600536810051743/om2378sup1.cif

Structure factors: contains datablocks I. DOI: 10.1107/S1600536810051743/om2378Isup2.hkl

Additional supplementary materials:  crystallographic information; 3D view; checkCIF report

## Figures and Tables

**Table 1:** Hydrogen-bond geometry (Å, °)

*D*—H⋯*A*	*D*—H	H⋯*A*	*D*⋯*A*	*D*—H⋯*A*
N1—H1⋯O1^i^	0.86	2.24	2.909 (2)	135
C6—H6⋯O1^i^	0.93	2.63	3.363 (2)	137
C10—H10⋯O2^ii^	0.98	2.36	3.255 (2)	152

Symmetry codes: (i) 
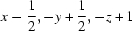
; (ii) 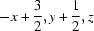.

**Fig. 1.**
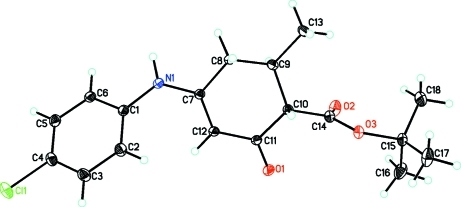
Diagram of tert-butyl- 4-(4-chlorophenlyamino)-6-methyl-2-oxocyclohex-3-enecarboxylate showing atom labeling scheme. Thermal ellipsoids drawn at the 30% probability level.

**Fig. 2.**
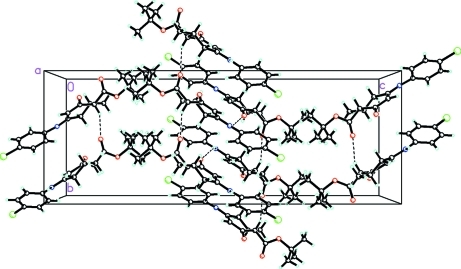
The molecular packing for tert-butyl- 4-(4-chlorophenlyamino)-6-methyl-2-oxocyclohex-3-enecarboxylate viewed down the a axis. Intermolecular interactions are shown by dashed lines.

## supplementary crystallographic information

### Comment

Our research on enaminones has led to several compounds possessing anticonvulsant properties (Edafiogho *et al.*, 1992; Eddington *et al.*, 2003; Scott *et al.*, 1993, 1995). The present work is part of a structural study of enaminones. Our group has extensively studied the effects of modification of the enaminone with substitutions at the methyl ester, ethyl ester, and without the ester group. We synthesized a series of carbo-*tert*-butoxy esters to evaluate the effect of added bulk and lipophilicity to the ester functionality. These compounds showed significant anticonvulsant activity. The title compound, *tert*-butyl- 4-(4-chlorophenlyamino)-6-methyl-2-oxocyclohex-3-enecarboxylate is highly active, with activity at <100 mg kg^-1^. The compound was active in maximal electroshock seizure evaluation (MES) in mice, indicative of protection against tonic-clonic convulsions in humans (1/4 rats protected at 1 h, 2 h and 4 h post dose at 50 mg kg dose). Toxicity tests showed no toxicity in rats (oral) up to 4 h at 50 mg/kg per dose. The MES study in mice showed 1/1 animals were protected at 300 mg/kg at 30 minutes. In 4 h testing, 3/3 animals were protected at 100 mg/kg and 1/1 animals protected at 300 mg/kg dose. In toxicity studies, at 15 min, 2/8 mice (ip = interperitoneal) showed toxicity at 500 mg/kg dose. A 2 h MES protection test in mice (ip) displayed 3/8 animals protected at 85 mg/kg dose, 4/8 animals protected at 100 mg/kg, and 7/8 animals were protected at 170 mg/kg (optimum dose). In mice, the MES ED50 (median effective dose) of 106.34 mg kg^-1^ and TD50 (median toxic dose) of 500 mg kg^-1^ TD, 95% confidence inteval. The scMET (subcuntaneous metrazole) test showed no protection at 250 or 500 mg/kg during a 2 h range.

In view of the therapeutic interest in this compound its structure was determined. The conformation adopted by the molecule is such that the dihedral angle between the phenyl ring and conjugated part of the enaminone ring is 55.19 (9)°. The ester substituent makes a dihedral angle of 81.0 (2)° with this latter moiety. The crystal structure is held together by strong N—H···O and weak C—H···O intermolecular interactions.

### Experimental

4-Carbo-*tert*-butoxy-5-methylcyclohexane-1,3-dione (6. 11 g, 27 mmol), mp 145–146° C (lit. mp 130–131.5°C),7 and 4-chloroaniline (4.21 g, 33 mmol) were added to a mixture of absolute EtOH (100 ml) and EtOAc (100 ml), and the solution was refluxed and stirred for 6 h. Evaporation under reduced pressure yielded a yellow solid which was recrystallized from 2-PrOH: yield 3.96 g (43%); mp 190–192° C; ^1^H NMR (CDC1_3_) 6 1.10 (3*H*, d, J = 6.3 Hz, CH_3_), 1.48 (9*H*, s, 3 *x* CH_3_ of *tert*-butyl group), 2.22–2.63 (3*H*, m, CH_2_ + CH of cyclohexene ring), 2.90 (lH, d, J =11.0 Hz, CHI, 5.45 (lH, 8, =CHI, 6.90 (lH, bs, NH), 7.05–7.30 (4*H*, m, C_6_H_4_).

### Refinement

H atoms were placed in geometrically idealized positions and constrained to ride on their parent atoms with a C—H distance of 0.93 and 0.98 Å *U*_iso_(H) = 1.2*U*_eq_(C) and 0.96 Å for CH_3_ [*U*_iso_(H) = 1.5*U*_eq_(C)]. The H atoms attached to N were idealized with an N–H distance of 0.86 Å.

### Crystal data

**Table:**

C_18_H_22_ClNO_3_	*D*_x_ = 1.262 Mg m^−^^3^
*M**_r_* = 335.82	Mo *K*α radiation, λ = 0.71073 Å
Orthorhombic, *P**b**c**a*	Cell parameters from 5335 reflections
*a* = 11.0801 (3) Å	θ = 4.5–77.5°
*b* = 10.9095 (3) Å	µ = 0.23 mm^−^^1^
*c* = 29.2474 (7) Å	*T* = 295 K
*V* = 3535.39 (16) Å^3^	Plate, colorless
*Z* = 8	0.45 × 0.38 × 0.08 mm
*F*(000) = 1424	

### Data collection

**Table:**

Oxford Diffraction Xcalibur Ruby Gemini diffractometer	3708 independent reflections
Radiation source: Enhance (Cu) X-ray Source	2925 reflections with *I* > 2σ(*I*)
graphite	*R*_int_ = 0.030
Detector resolution: 10.5081 pixels mm^-1^	θ_max_ = 26.8°, θ_min_ = 2.3°
ω scans	*h* = −13→14
Absorption correction: multi-scan (*CrysAlis PRO*; Oxford Diffraction, 2007)	*k* = −13→7
*T*_min_ = 0.93, *T*_max_ = 0.98	*l* = −22→36
9798 measured reflections	

### Refinement

**Table:**

Refinement on *F*^2^	Primary atom site location: structure-invariant direct methods
Least-squares matrix: full	Secondary atom site location: difference Fourier map
*R*[*F*^2^ > 2σ(*F*^2^)] = 0.055	Hydrogen site location: inferred from neighbouring sites
*wR*(*F*^2^) = 0.172	H-atom parameters constrained
*S* = 1.09	*w* = 1/[σ^2^(*F*_o_^2^) + (0.1062*P*)^2^ + 0.3243*P*] where *P* = (*F*_o_^2^ + 2*F*_c_^2^)/3
3708 reflections	(Δ/σ)_max_ < 0.001
212 parameters	Δρ_max_ = 0.39 e Å^−^^3^
0 restraints	Δρ_min_ = −0.25 e Å^−^^3^

### Special details

**Table:**

Geometry. All e.s.d.'s (except the e.s.d. in the dihedral angle between two l.s. planes) are estimated using the full covariance matrix. The cell e.s.d.'s are taken into account individually in the estimation of e.s.d.'s in distances, angles and torsion angles; correlations between e.s.d.'s in cell parameters are only used when they are defined by crystal symmetry. An approximate (isotropic) treatment of cell e.s.d.'s is used for estimating e.s.d.'s involving l.s. planes.
Refinement. Refinement of *F*^2^ against ALL reflections. The weighted *R*-factor *wR* and goodness of fit *S* are based on *F*^2^, conventional *R*-factors *R* are based on *F*, with *F* set to zero for negative *F*^2^. The threshold expression of *F*^2^ > σ(*F*^2^) is used only for calculating *R*-factors(gt) *etc*. and is not relevant to the choice of reflections for refinement. *R*-factors based on *F*^2^ are statistically about twice as large as those based on *F*, and *R*- factors based on ALL data will be even larger.

### Fractional atomic coordinates and isotropic or equivalent isotropic displacement parameters (Å^2^)

**Table:**

	*x*	*y*	*z*	*U*_iso_*/*U*_eq_	
Cl1	0.91865 (8)	0.65446 (7)	0.66441 (3)	0.0944 (3)	
O1	0.91038 (13)	0.17651 (17)	0.43476 (6)	0.0689 (4)	
O2	0.7403 (2)	0.01470 (14)	0.37719 (6)	0.0785 (5)	
O3	0.78836 (13)	0.16309 (12)	0.32703 (4)	0.0545 (3)	
N1	0.63017 (13)	0.40873 (17)	0.52106 (5)	0.0539 (4)	
H1	0.5536	0.4180	0.5243	0.065*	
C1	0.70410 (16)	0.46315 (18)	0.55484 (6)	0.0492 (4)	
C2	0.8068 (2)	0.5287 (2)	0.54365 (7)	0.0628 (5)	
H2	0.8314	0.5333	0.5133	0.075*	
C3	0.8732 (2)	0.5875 (2)	0.57713 (8)	0.0682 (6)	
H3	0.9429	0.6305	0.5695	0.082*	
C4	0.8349 (2)	0.5817 (2)	0.62193 (7)	0.0621 (5)	
C5	0.7323 (2)	0.5180 (2)	0.63334 (7)	0.0659 (5)	
H5	0.7068	0.5154	0.6636	0.079*	
C6	0.66736 (17)	0.4583 (2)	0.60016 (6)	0.0584 (5)	
H6	0.5985	0.4144	0.6081	0.070*	
C7	0.66666 (15)	0.34364 (17)	0.48417 (5)	0.0447 (4)	
C8	0.56893 (15)	0.32234 (19)	0.44950 (6)	0.0507 (4)	
H8A	0.5601	0.3956	0.4310	0.061*	
H8B	0.4933	0.3091	0.4654	0.061*	
C9	0.59232 (15)	0.21420 (18)	0.41826 (6)	0.0489 (4)	
H9	0.5873	0.1391	0.4365	0.059*	
C10	0.72128 (15)	0.22487 (16)	0.39930 (6)	0.0447 (4)	
H10A	0.7270	0.3016	0.3820	0.054*	
C11	0.81263 (14)	0.23012 (17)	0.43840 (6)	0.0472 (4)	
C12	0.78047 (15)	0.29974 (18)	0.47760 (6)	0.0485 (4)	
H12	0.8391	0.3159	0.4995	0.058*	
C13	0.49731 (19)	0.2070 (2)	0.38044 (7)	0.0635 (5)	
H13A	0.4184	0.2022	0.3939	0.095*	
H13B	0.5115	0.1355	0.3621	0.095*	
H13C	0.5024	0.2789	0.3616	0.095*	
C14	0.75164 (18)	0.12016 (16)	0.36732 (6)	0.0507 (4)	
C15	0.8185 (2)	0.0796 (2)	0.28902 (6)	0.0586 (5)	
C16	0.9248 (3)	0.0025 (4)	0.30166 (10)	0.1008 (11)	
H16C	0.9005	−0.0585	0.3234	0.151*	
H16D	0.9863	0.0534	0.3149	0.151*	
H16A	0.9560	−0.0368	0.2748	0.151*	
C17	0.8512 (4)	0.1665 (3)	0.25068 (9)	0.0955 (10)	
H17A	0.9144	0.2205	0.2607	0.143*	
H17B	0.7816	0.2139	0.2423	0.143*	
H17C	0.8783	0.1204	0.2247	0.143*	
C18	0.7086 (3)	0.0050 (4)	0.27656 (11)	0.1025 (11)	
H18A	0.6868	−0.0465	0.3019	0.154*	
H18B	0.7264	−0.0450	0.2504	0.154*	
H18C	0.6428	0.0590	0.2695	0.154*	

### Atomic displacement parameters (Å^2^)

**Table:**

	*U*^11^	*U*^22^	*U*^33^	*U*^12^	*U*^13^	*U*^23^
Cl1	0.1084 (6)	0.0928 (5)	0.0821 (4)	−0.0154 (4)	−0.0342 (4)	−0.0178 (4)
O1	0.0460 (7)	0.0958 (11)	0.0648 (9)	0.0202 (7)	−0.0022 (6)	−0.0154 (8)
O2	0.1255 (15)	0.0446 (8)	0.0653 (9)	0.0057 (8)	0.0129 (9)	0.0027 (7)
O3	0.0694 (8)	0.0495 (7)	0.0446 (6)	0.0010 (6)	0.0015 (6)	−0.0036 (5)
N1	0.0414 (7)	0.0721 (10)	0.0481 (8)	0.0038 (7)	0.0036 (6)	−0.0087 (7)
C1	0.0474 (8)	0.0557 (10)	0.0443 (8)	0.0047 (7)	0.0002 (6)	−0.0028 (7)
C2	0.0743 (13)	0.0642 (12)	0.0499 (10)	−0.0136 (10)	0.0051 (9)	0.0020 (9)
C3	0.0743 (13)	0.0604 (12)	0.0700 (12)	−0.0195 (11)	−0.0015 (10)	0.0011 (10)
C4	0.0714 (12)	0.0577 (11)	0.0571 (10)	0.0041 (9)	−0.0161 (9)	−0.0065 (9)
C5	0.0642 (11)	0.0887 (15)	0.0447 (9)	0.0067 (11)	−0.0011 (8)	−0.0058 (10)
C6	0.0470 (9)	0.0802 (13)	0.0479 (9)	0.0015 (9)	0.0032 (7)	−0.0037 (9)
C7	0.0413 (8)	0.0528 (9)	0.0399 (7)	−0.0010 (7)	0.0017 (6)	0.0020 (7)
C8	0.0364 (7)	0.0677 (11)	0.0480 (8)	0.0020 (7)	−0.0013 (6)	−0.0027 (8)
C9	0.0441 (8)	0.0538 (10)	0.0489 (9)	−0.0056 (7)	−0.0048 (7)	0.0038 (7)
C10	0.0476 (8)	0.0448 (8)	0.0418 (8)	−0.0005 (6)	−0.0001 (6)	0.0007 (7)
C11	0.0383 (7)	0.0561 (9)	0.0471 (8)	0.0006 (7)	0.0020 (6)	0.0001 (7)
C12	0.0378 (7)	0.0630 (10)	0.0445 (8)	0.0029 (7)	−0.0034 (6)	−0.0022 (7)
C13	0.0538 (10)	0.0735 (13)	0.0633 (11)	−0.0086 (9)	−0.0151 (9)	−0.0045 (10)
C14	0.0575 (9)	0.0469 (9)	0.0478 (9)	0.0033 (8)	−0.0038 (7)	−0.0005 (7)
C15	0.0693 (11)	0.0613 (11)	0.0451 (9)	0.0034 (9)	−0.0008 (8)	−0.0103 (8)
C16	0.102 (2)	0.126 (3)	0.0742 (16)	0.051 (2)	0.0111 (15)	−0.0059 (17)
C17	0.147 (3)	0.0849 (17)	0.0544 (13)	−0.0033 (19)	0.0192 (16)	−0.0025 (12)
C18	0.102 (2)	0.125 (3)	0.0800 (17)	−0.0311 (19)	0.0000 (15)	−0.0427 (18)

### Geometric parameters (Å, °)

**Table:**

Cl1—C4	1.742 (2)	C9—C13	1.529 (2)
O1—C11	1.235 (2)	C9—C10	1.537 (2)
O2—C14	1.193 (3)	C9—H9	0.9800
O3—C14	1.332 (2)	C10—C14	1.514 (2)
O3—C15	1.475 (2)	C10—C11	1.528 (2)
N1—C7	1.353 (2)	C10—H10A	0.9800
N1—C1	1.414 (2)	C11—C12	1.421 (2)
N1—H1	0.8600	C12—H12	0.9300
C1—C2	1.383 (3)	C13—H13A	0.9600
C1—C6	1.388 (3)	C13—H13B	0.9600
C2—C3	1.383 (3)	C13—H13C	0.9600
C2—H2	0.9300	C15—C16	1.494 (3)
C3—C4	1.379 (3)	C15—C18	1.509 (3)
C3—H3	0.9300	C15—C17	1.512 (3)
C4—C5	1.373 (3)	C16—H16C	0.9600
C5—C6	1.373 (3)	C16—H16D	0.9600
C5—H5	0.9300	C16—H16A	0.9600
C6—H6	0.9300	C17—H17A	0.9600
C7—C12	1.363 (2)	C17—H17B	0.9600
C7—C8	1.502 (2)	C17—H17C	0.9600
C8—C9	1.515 (3)	C18—H18A	0.9600
C8—H8A	0.9700	C18—H18B	0.9600
C8—H8B	0.9700	C18—H18C	0.9600
			
C14—O3—C15	121.26 (15)	C11—C10—H10	108.1
C7—N1—C1	127.17 (15)	C9—C10—H10	108.1
C7—N1—H1	116.4	O1—C11—C12	122.87 (17)
C1—N1—H1	116.4	O1—C11—C10	119.88 (16)
C2—C1—C6	119.15 (18)	C12—C11—C10	117.24 (15)
C2—C1—N1	121.90 (17)	C7—C12—C11	122.28 (16)
C6—C1—N1	118.77 (17)	C7—C12—H12	118.9
C3—C2—C1	120.67 (19)	C11—C12—H12	118.9
C3—C2—H2	119.7	C9—C13—H13A	109.5
C1—C2—H2	119.7	C9—C13—H13B	109.5
C4—C3—C2	119.2 (2)	H13A—C13—H13B	109.5
C4—C3—H3	120.4	C9—C13—H13C	109.5
C2—C3—H3	120.4	H13A—C13—H13C	109.5
C5—C4—C3	120.60 (19)	H13B—C13—H13C	109.5
C5—C4—Cl1	119.86 (17)	O2—C14—O3	125.85 (18)
C3—C4—Cl1	119.54 (19)	O2—C14—C10	123.68 (18)
C6—C5—C4	120.15 (19)	O3—C14—C10	110.43 (15)
C6—C5—H5	119.9	O3—C15—C16	109.85 (18)
C4—C5—H5	119.9	O3—C15—C18	109.42 (19)
C5—C6—C1	120.22 (19)	C16—C15—C18	113.1 (3)
C5—C6—H6	119.9	O3—C15—C17	103.07 (18)
C1—C6—H6	119.9	C16—C15—C17	110.3 (2)
N1—C7—C12	124.99 (16)	C18—C15—C17	110.6 (2)
N1—C7—C8	113.84 (15)	C15—C16—H16C	109.5
C12—C7—C8	121.18 (15)	C15—C16—H16D	109.5
C7—C8—C9	113.86 (15)	H16C—C16—H16D	109.5
C7—C8—H8A	108.8	C15—C16—H16A	109.5
C9—C8—H8A	108.8	H16C—C16—H16A	109.5
C7—C8—H8B	108.8	H16D—C16—H16A	109.5
C9—C8—H8B	108.8	C15—C17—H17A	109.5
H8A—C8—H8B	107.7	C15—C17—H17B	109.5
C8—C9—C13	111.01 (16)	H17A—C17—H17B	109.5
C8—C9—C10	108.52 (14)	C15—C17—H17C	109.5
C13—C9—C10	112.52 (16)	H17A—C17—H17C	109.5
C8—C9—H9	108.2	H17B—C17—H17C	109.5
C13—C9—H9	108.2	C15—C18—H18A	109.5
C10—C9—H9	108.2	C15—C18—H18B	109.5
C14—C10—C11	110.08 (14)	H18A—C18—H18B	109.5
C14—C10—C9	111.85 (15)	C15—C18—H18C	109.5
C11—C10—C9	110.40 (14)	H18A—C18—H18C	109.5
C14—C10—H10	108.1	H18B—C18—H18C	109.5
			
C7—N1—C1—C2	44.5 (3)	C8—C9—C10—C11	57.46 (19)
C7—N1—C1—C6	−140.4 (2)	C13—C9—C10—C11	−179.30 (17)
C6—C1—C2—C3	0.8 (3)	C14—C10—C11—O1	17.6 (2)
N1—C1—C2—C3	175.9 (2)	C9—C10—C11—O1	141.56 (18)
C1—C2—C3—C4	−1.0 (4)	C14—C10—C11—C12	−163.43 (16)
C2—C3—C4—C5	0.2 (4)	C9—C10—C11—C12	−39.5 (2)
C2—C3—C4—Cl1	179.48 (19)	N1—C7—C12—C11	178.91 (18)
C3—C4—C5—C6	0.7 (4)	C8—C7—C12—C11	−1.1 (3)
Cl1—C4—C5—C6	−178.60 (18)	O1—C11—C12—C7	−170.34 (19)
C4—C5—C6—C1	−0.8 (3)	C10—C11—C12—C7	10.7 (3)
C2—C1—C6—C5	0.1 (3)	C15—O3—C14—O2	0.7 (3)
N1—C1—C6—C5	−175.1 (2)	C15—O3—C14—C10	−177.35 (16)
C1—N1—C7—C12	12.9 (3)	C11—C10—C14—O2	70.1 (3)
C1—N1—C7—C8	−167.05 (18)	C9—C10—C14—O2	−53.0 (3)
N1—C7—C8—C9	−158.30 (16)	C11—C10—C14—O3	−111.80 (17)
C12—C7—C8—C9	21.7 (3)	C9—C10—C14—O3	125.08 (17)
C7—C8—C9—C13	−173.31 (16)	C14—O3—C15—C16	−64.0 (3)
C7—C8—C9—C10	−49.2 (2)	C14—O3—C15—C18	60.7 (3)
C8—C9—C10—C14	−179.60 (14)	C14—O3—C15—C17	178.4 (2)
C13—C9—C10—C14	−56.4 (2)		

### Hydrogen-bond geometry (Å, °)

**Table:**

*D*—H···*A*	*D*—H	H···*A*	*D*···*A*	*D*—H···*A*
N1—H1···O1^i^	0.86	2.24	2.909 (2)	135.
C6—H6···O1^i^	0.93	2.63	3.363 (2)	137.
C10—H10···O2^ii^	0.98	2.36	3.255 (2)	152.0

Symmetry codes: (i) *x*−1/2, −*y*+1/2, −*z*+1; (ii) −*x*+3/2, *y*+1/2, *z*.
